# Alternative Treatments to Exercise for the Attenuation of Disuse-Induced Skeletal Muscle Atrophy in Rats

**DOI:** 10.3390/muscles3030020

**Published:** 2024-07-22

**Authors:** Jinho Park, T. Brock Symons, Eun Hye Kwon, Eunhee Chung, Sukho Lee

**Affiliations:** 1 Department of Exercise Rehabilitation, Gachon University, Incheon 21936, Republic of Korea; jinho04oo@gmail.com; 2 Department of Counselling, Health, and Kinesiology, Texas A&M University-San Antonio, San Antonio, TX 78224, USA; tsymons@tamusa.edu (T.B.S.); ekwon@tamusa.edu (E.H.K.); 3 Department of Kinesiology, University of Texas San Antonio, San Antonio, TX 78249, USA; eunhee.chung@utsa.edu

**Keywords:** acupuncture, electro-acupuncture, electrical stimulation, immobilization, cast, MAFbx, MuRF1, disuse atrophy, muscle wasting, gastrocnemius

## Abstract

The prevalence of skeletal muscle atrophy, caused by disease and aging, is rising as life expectancy increases. Exercise is the most effective treatment option; however, it is often impractical for individuals suffering from disease or bedridden. The formulation of non-exercise-based interventions is necessary. This study assessed the impact of acupuncture (AC), electro-acupuncture (EA), and electrical stimulation (ES) on muscle mass and contractile properties in a model of casting-induced muscle atrophy. Sprague-Dawley rats (n = 40) were assigned to five groups: control (CON), cast (CT), cast receiving AC (CT-AC), cast receiving EA (CT-EA), and cast receiving ES (CT-ES) (n = 8 each). Treatments were 15 min and three times/week for 14 days. Contractile properties and protein markers of atrophy and inflammation were measured. Casting decreased muscle mass and fiber cross-sectional area, but AC, EA, and ES attenuated cast-induced muscle atrophy. All treatments increased peak twitch tension compared to CT. CT increased the protein levels of MAFbx and MuRF1, while AC, EA, and ES mitigated the elevation of these proteins. Our results indicate that acupuncture, electro-acupuncture, and electrical stimulation show promise as therapeutic strategies to counteract skeletal muscle loss and dysfunction resulting from disuse atrophy caused by injury, disease, and aging.

## 1. Introduction

As the global population continues to age rapidly, there has been a simultaneous increase in the number of patients with musculoskeletal disorders [1]. Skeletal muscles account for approximately 40 to 50% of body weight. They are responsible for essential functions such as energy metabolism, body temperature maintenance, respiration, and postural maintenance [2,3,4]. As individuals age, there are significant decreases in muscle mass, leading to changes in body composition [5,6,7]. Muscle atrophy, characterized by a reduction in muscle size, is the result of muscle tissue wasting due to factors such as a sedentary lifestyle, aging, or disuse. This condition leads to a loss of nerve control and a significant decrease in the capacity for force production [8].

The importance of preventive methods and treatment for muscle atrophy is receiving worldwide attention from different fields. The decline of skeletal muscle mass is a significant concern for individuals who are unable to engage in physical activity due to factors such as being restricted to bed or having cast-immobilization. This is because the lack of mechanical loading experienced by skeletal muscle exacerbates skeletal muscle atrophy. Mechanical loading plays a vital role in maintaining skeletal muscle mass. However, there are situations where traditional forms of physical activity, like resistance exercise, may not be possible due to medical conditions relating to physiology and pathophysiology. Hence, it is crucial to explore different treatments that can effectively prevent or attenuate the loss of skeletal muscle during periods of skeletal muscle disuse. Acupuncture has been an essential traditional medicine in East Asian countries since ancient times [9]. Its purpose is to stimulate the nervous system and treat pain by inserting a fine needle in specific parts of the body [10,11,12]. Various studies have reported positive effects of acupuncture for treating skeletal muscle atrophy and musculoskeletal disorders [13,14,15,16]. Numerous studies have shown beneficial outcomes of electro-acupuncture in the treatment of skeletal muscle atrophy and musculoskeletal disorders as well [13,17]. Electro-acupuncture is a form of acupuncture therapy that involves the use of electrical stimulation applied to acupuncture needles. It combines traditional acupuncture techniques with modern technology to enhance the therapeutic effects of the treatment. On the other hand, electrical stimulation is mainly used within the physical therapy field for patients with musculoskeletal disorders or cardiovascular diseases, especially post-stroke [18,19]. Electrical stimulation triggers the body’s physiological response and nerve impulses, promotes blood circulation, treats pain, and strengthens muscles [20]. The study showed that applying electric stimulation at a certain frequency (1 Hz–100 Hz) causes maximum muscle contraction in Type I and Type II muscle fibers. Electrical stimulation has demonstrated positive effects on muscle strength [21]. However, it is still uncertain whether acupuncture alone or when combined with electrical stimulation is effective in treating skeletal muscle atrophy and musculoskeletal disorders. This study aimed to compare the impact of acupuncture (AC), electro-acupuncture (EA), and electrical stimulation (ES) on muscle mass and contractile properties in a model of casting-induced muscle atrophy. We hypothesized that acupuncture, electrical stimulation, and electro-acupuncture would attenuate muscle atrophy induced by disuse.

## 2. Methods

### 2.1. Animals

Nine-week female Sprague-Dawley rats (n = 40) weighing 205 ± 11 g were obtained from Charles River (Stone Ridge, NY, USA). Animals were kept on a 12-h light-dark cycle (22.8 ± 0.3 °C) and given free access to food and water. Animals were randomly assigned to five groups: no cast (CON, n = 8); cast (CT, n = 8); cast plus acupuncture treatment (CT-AC, n = 8); cast plus electro-acupuncture treatment (CT-EA, n = 8); and cast plus electrical stimulation treatment (CT-ES, n = 8). This study was approved by the Institutional Animal Care and Use Committee at Texas A&M University-San Antonio (protocol reference no. 2020-06).

### 2.2. Cast-Immobilization

Before casting, animals were anesthetized (isoflurane gas, 1–2 L/min). To reduce abrasion, the hind limb was covered with pre-wrap (Johnson & Johnson, Skillman, NJ, USA). To induce atrophy of the gastrocnemius muscle of the left hind limb, a cast (made of plaster of Paris, Craft Wrap™, Goja, Miami, FL, USA) extending 20 mm above the patella was used to position the ankle joint in plantar flexion. Bitter apple spray (Grannick’s Bitter Apple, Norwalk, CT, USA) was applied to minimize the animals’ tendency to chew or damage the cast. Following the casting of the animals, it was confirmed that they were able to move freely about their cages and had free access to food and water. The animals’ hind limbs were examined daily for trauma and swelling, along with the cast for damage, during the 14-day casting period.

### 2.3. Treatment

**Figure 1:**
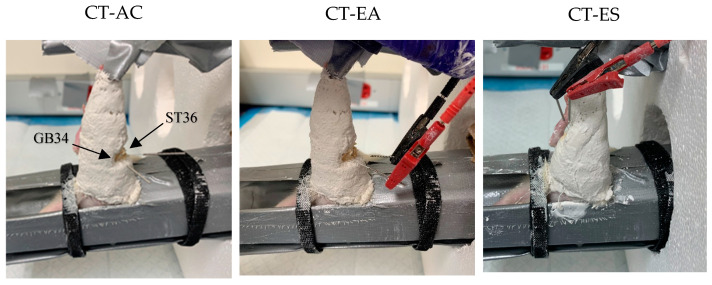
Stomach 36 (ST36) and Gallbladder 34 (GB34) needle position. CT-AC: acupuncture, CT-EA: electro-acupuncture, and CT-ES: electrical stimulation.

The acupuncture treatment consisted of inserting 15 mm needles with a diameter of 0.16 mm (DBC™ Spring Ten, Lhasa OMS, Weymouth, MA, USA) to an approximate depth of 0.3–0.5 mm into two acupuncture points (ST36 and GB34) on the left hind limb of the animal. Acupoint ST36 was located inferior to the knee and lateral to the lateral border of the tibial tuberosity, while the location of acupoint GB34 was found in the depression anterior and inferior to the fibular head (Figure 1). Acupuncture was administered six times (3 x/week) across the 14-day casting period, with each session lasting 15 min.

Electro-acupuncture was administered through the same acupoints, ST36 and GB34, as those used in the acupuncture intervention (Figure 1). A constant current electrical stimulator (ES-160, ITOCO, Nerima-Ku, Tokyo, Japan) supplied a 6.4 mA continuous waveform (10 Hz, phase duration 150 µs) to the needles at ST36 and GB34. The electro-acupuncture intervention was administered six times (3 x/week) across the 14-day casting period, with each session lasting 15 min.

Electrical stimulation was delivered to the gastrocnemius skeletal muscle via needle electrodes placed in the muscle belly (Figure 1) and utilized the aforementioned electrical stimulation protocol. Electrical stimulation was administered six times (3 x/week) across the 14-day casting period, with each session lasting 15 min.

### 2.4. Contractile Properties, Muscle Characteristics, and Protein Measures

Contractile properties were assessed 24 h after the completion of the casting period. Animals were anesthetized using isoflurane gas (1–2 L/min), ketamine (80 mg/kg), and xylazine (8 mg/kg). The contractile properties of the gastrocnemius skeletal muscle were measured using a dual-mode servo and galvanometer (model 310C, Aurora Scientific, Richmond, ON, Canada). LabVIEW software (Version 3.0, National Instruments Corp., Austin, TX, USA) was used to analyze the data.

The sciatic nerve was stimulated with a silver wire electrode (Isolated Pulse Stimulator, Model 2100, A-M System Inc., Sequim, WA, USA) to activate the gastrocnemius muscle. A single twitch at 0.5 Hz and seven volts, with a pulse duration of half a millisecond, was used to measure peak twitch tension (Pt), contraction time, and half-relaxation time (1/2 Rt) [22]. The determination of peak tetanic tension (Po) involved applying a single 330 ms train at 14 V. To increase the intensity of contraction, the frequency of stimulation was gradually increased from an initial frequency of 100 Hz until the Po reached a plateau [23]. 

After contractile property measurement, the animals were euthanized via intracardiac injection of ketamine (80 mg/kg) and xylazine (8 mg/kg). The gastrocnemius skeletal muscle was removed and stored at −80 °C.

Gastrocnemius muscle cross-sectional area (CSA) was determined using the following formula: CSA (mm^2^) = muscle mass (mg)/[1.06 (mg/mm^3^) * muscle length (mm)]. Muscle fiber length (Lo) was approximated using the following formula: Lo (mm) = muscle mass length (mm) * 0.25 [23]. 

The gastrocnemius muscles were homogenized in a RIPA buffer containing protease and phosphatase inhibitors, then spun at 14,000 rcf for 30 min at 4 °C. Protein concentrations were quantified using a bicinchoninic acid assay. Western blot analysis was used to quantify muscle atrophy F-box (MAFbx) and muscle RING finger 1 (MuRF1) protein levels.

Muscle atrophy F-box and MuRF1 protein levels were determined via Western blot analysis. The preparation of skeletal muscle specimens, blocking, and antibody incubation procedures, as well as primary [MAFbx (SC-166806, 1:1000, Santa Cruz Biotechnology, Santa Cruz, CA, USA) and MuRF1 (SC-398608, 1:1000, Santa Cruz Biotechnology, Santa Cruz, CA, USA)] and secondary antibodies, have been previously described by Symons et al. [17]. 

Enzyme-linked immunosorbent assays (ELISA) (RayBiotech, Peachtree Corners, GA, USA) were used to measure interleukin-1beta (IL-1β), IL-6, monocyte chemoattractant protein-1 (MCP-1), and tumor necrosis factor-alpha (TNF-α) from plasma. All kits used reported published successful results with plasma samples. Sample dilutions were prepared using manufacturer suggestions, and values are expressed as pg/mL or ng/mL. 

Gastrocnemius skeletal muscle sections were frozen in an optimal cutting temperature compound (Scigen Tissue-Plus™ O.C.T. Compound, Fisher Health Care, Houston, TX, USA) following extraction. Sections (20 µm) were mounted and dried for 24 h. Gastrocnemius muscle sections were stained with hematoxylin and eosin (H&E) according to standard protocol and observed under X20 magnification (Leica Biosystem, Deer Park, IL, USA). Muscle fiber CSA was determined by averaging the CSA of 20 skeletal muscle fibers per animal using the Image J program (National Institutes of Health, Bethesda, MD, USA) [17].

### 2.5. Statistical Analysis

Data are presented as means ± standard deviations. IBM SPSS Statistics (version 27; IBM Corp, Armonk, NY, USA) was used for statistical analysis. Outcome measures were examined using separate one-way analyses of variance. Significant between-group effects were evaluated with a least significant difference test. Statistical significance was set at α = 0.05. 

## 3. Results

### 3.1. Animal Characteristics

Prior to casting, body weight did not differ between groups (*p* = 0.26), nor did it differ after the cast-immobilization period (14 days; *p* = 0.05; Table 1). Body weight was significantly increased (4.9%) over the cast-immobilization period (14 days) in the control group, while all treatment groups demonstrated a non-significant change in body weight (*p* > 0.05).

### 3.2. Muscle Characteristics

Fourteen days of cast-immobilization significantly reduced gastrocnemius muscle weights in all treatment groups compared to the CON group (*p* < 0.01). The CT-EA (−34%) and CT-ES (−35%) groups exhibited attenuated losses in gastrocnemius muscle weight, which were not different from those observed in the CT group (−42%). The CT-AC group demonstrated the least amount of loss in gastrocnemius muscle weight (−31%) and was significantly higher than the CT group (*p* = 0.02; Table 2). The ratio of gastrocnemius muscle weight to body weight was employed as a determinant of muscle atrophy, and 14 days of cast-immobilization demonstrated a group effect (*p* <0.01). All treatment groups exhibited reduced ratios of gastrocnemius muscle weight to body weight in comparison to the CON group (*p* < 0.01). The CT-AC, CT-EA, and CT-ES groups exhibited attenuated gastrocnemius muscle weight to body weight ratios; however, they did not differ from the CT group. Muscle length and whole gastrocnemius muscle CSA did not differ amongst the groups. The CSA of gastrocnemius muscle fibers differed among the three treatment groups (*p* < 0.01). The CT group demonstrated a 58% reduction in gastrocnemius muscle fiber CSA compared to the CON group (*p* < 0.01). In comparison to the CON group, the CT-AC, CT-EA, and CT-ES groups displayed a 48%, 50%, and 48% reduction in muscle fiber CSA, respectively (*p* < 0.01). Further, when compared to the CT group, gastrocnemius muscle fiber CSA for the CT-AC, CT-EA, and CT-ES groups was significantly greater (*p* < 0.01).

### 3.3. Contractile Properties of the Gastrocnemius Muscle

Contraction time was significantly higher in the CT-AC, CT-EA, and CT-ES groups compared to the CON group (*p* < 0.05), and half-relaxation time was significantly higher in the CT-EA group than the CT group (*p* < 0.05) following the 14-day cast-immobilization period (Table 3). Peak twitch tension was reduced by 44% in the CT group compared to the CON group after 14 days of cast-immobilization. Peak twitch tension was significantly higher in the CT-AC (26.7%), CT-EA (25.9%), and CT-ES (31.2%) groups compared to the CT groups (*p* < 0.05) following 14 days of cast-immobilization. Compared to the CON group, peak tetanic tension was significantly lower in the CT group, while the treatment groups did not differ following cast-immobilization for specific peak tetanic tension.

### 3.4. Biochemical Measures

**Figure 2:**
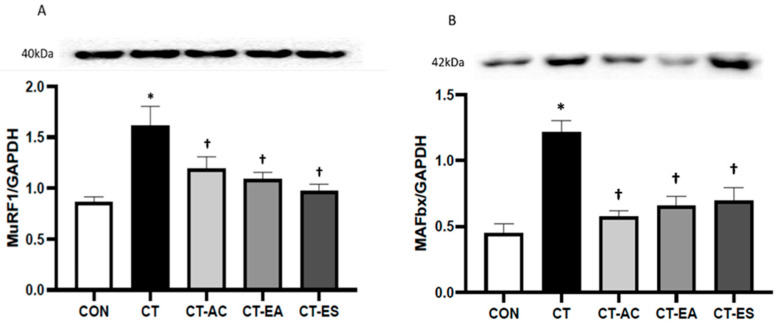
(**A**) Change in muscle RING finger 1 (MuRF1) and (**B**) muscle atrophy F-box (MAFbx) protein levels. CON = control; CAST = cast; CT-AC = cast + acupuncture; CT-EA = cast + electro-acupuncture; CT-ES = cast + electrical stimulation. * Difference from CON group (*p* < 0.05). † Difference from CT group (*p* < 0.05). Values are mean and standard deviation.

Cast immobilization (for 14 days) resulted in a significant group effect (*p* < 0.01) on MuRF1 protein levels. Cast immobilization significantly elevated MuRF1 protein levels within the gastrocnemius muscle (CON: 0.9 ± 0.2 vs. CT: 1.6 ± 0.5; *p* < 0.01) (Figure 2). In comparison to the CT group, the three treatment groups (CT-AC, CT-EA, and CT-ES) showed decreased MuRF1 protein levels (CT: 1.6 ± 0.5 vs. CT-AC: 1.2 ± 0.3, CT-EA: 1.1 ± 0.2, CT-ES: 1.0 ± 0.2; *p* < 0.05).

Regarding MAFbx protein levels, cast-immobilization for 14 days generated a significant group effect (*p* < 0.01). Compared to the CON group, MAFbx levels significantly increased in the CT group (0.5 ± 0.2 vs. 1.2 ± 0.3; *p* < 0.01) (Figure 2). In comparison to the CT group, the three treatment groups exhibited significantly reduced MAFbx protein levels (CT: 1.2 ± 0.3 vs. CT-AC: 0.6 ± 0.1, CT-EA: 0.7 ± 0.2, and CT-ES: 0.7 ± 0.3; *p* < 0.02).

Our study found no significant differences in IL-6 levels (pg/mL) among the groups: 29.43 ± 2.93 (n = 6) in CON, 31.96 ± 3.03 (n = 7) in CT, 32.88 ± 3.86 (n = 7) in CT-AC, 31.85 ± 3.62 (n = 7) in CT-ES, and 29.86 ± 2.02 (n = 8) in CT-EA. The low detectability of IL-1β, TNF-α, and MCP-1 in the vast majority of samples limited the interpretation of the results. Consequently, further investigation into the inflammatory pathways potentially contributing to muscle atrophy was not pursued within the context of this study.

## 4. Discussion

Loss of skeletal muscle mass can impair an individual’s recovery process by diminishing functional capacity and potentially prolonging the healing period after limb immobilization. We hypothesized that the three treatments would significantly reduce disuse skeletal muscle atrophy and the decline in skeletal muscle contractile function. It was determined that acupuncture, electro-acupuncture, and electrical stimulation significantly attenuated protein markers of skeletal muscle disuse atrophy when administered during the cast-immobilization period of 14 days. The levels of MuRF1 and MAFbx proteins were significantly attenuated by acupuncture, electro-acupuncture, and electrical stimulation compared to the CT group (ranging from 26% to 53%). Additionally, CT-AC, CT-EA, and CT-ES were found to significantly attenuate muscle fiber CSA loss in the gastrocnemius compared to the CT group. Lastly, this study demonstrated that CT-AC, CT-EA, and CT-ES during the cast-immobilization for 14 days preserved peak twitch and tetanic tensions when compared to the CON group.

### 4.1. Muscle Characteristics

Skeletal muscle atrophy was quantified through alterations in gastrocnemius muscle weight and the ratio between gastrocnemius muscle weight and body weight. Cast immobilization (for 14 days) reduced both parameters (*p* < 0.05), consistent with previous results [17,24,25]. Among the current study treatment groups, only the CT-AC group differed from the CT group, demonstrating an 18% increase in muscle weight (*p* < 0.05). Su et al. (2016) demonstrated a 23% increase in mice following 15 min of low-frequency electro-acupuncture over two weeks of denervation-induced atrophy of the gastrocnemius muscle compared to denervation alone [24]. Symons et al. found no difference in plantaris muscle weight in rats following two weeks of cast-immobilization plus acupuncture compared to cast-immobilization alone [17]. Regarding the muscle weight-to-body weight ratio, Su et al.’s results differ from the current study, as they displayed a significant increase in the denervation-low-frequency electro-acupuncture group over denervation alone [24]. The current study and Symons et al. found no treatment effect (CT-AC, CT-EA, and CT-ES) versus cast immobilization. Methodological differences between the current study, manual acupuncture versus electro-acupuncture, and/or the muscle investigated may possibly explain the differences in results between studies [17]. The CT-AC group finding indicates the potential use of acupuncture as a supplementary treatment to help mitigate skeletal muscle wasting. 

Skeletal muscle fiber cross-sectional area is crucial for skeletal muscle function as it is directly associated with both the fiber’s and the whole muscle’s ability to generate force, power, and overall functional performance. The current study found a 58% decrease in skeletal muscle fiber CSA following cast immobilization for 14 days in all CT groups. This finding aligns with others observed across various skeletal muscles [17,26,27,28]. The present study demonstrated that applying six treatments over the 14-day casting period, including acupuncture, electro-acupuncture, and electrical stimulation, significantly diminished the loss of gastrocnemius muscle fiber cross-sectional area compared to the CT group. This finding aligns with previous research, which showed a significantly reduced muscle fiber cross-sectional area in the plantaris and soleus with the use of acupuncture, electro-acupuncture, and electrical stimulation compared to cast-immobilization [13,17]. However, in both previous studies and the current one, the level of improvement provided by the treatments was not sufficient to restore the CSA of the muscle fibers to the level of the control group. In contrast, Onda et al., employing 14 treatments of acupuncture and electro-acupuncture over two weeks of hind limb suspension in mice, demonstrated a non-significant reduction in gastrocnemius muscle fiber CSA [13]. This discrepancy in results may be attributed to the method of disuse employed, hind limb suspension versus cast-immobilization. Previous work indicates that hind limb suspension results in greater atrophy of slow-twitch muscles (e.g., soleus), while casting can produce equal levels of skeletal muscle loss in slow- and fast-twitch muscles (e.g., gastrocnemius/plantaris) [29]. These results demonstrate the promise of acupuncture, as well as electro-acupuncture and electrical stimulation, as alternatives to drug- and exercise-focused interventions in the management of skeletal muscle disuse atrophy.

### 4.2. Contractile Properties of the Gastrocnemius Muscle

Cast immobilization, coupled with the subsequent decrease in skeletal muscle fiber cross-sectional area, can have detrimental effects on skeletal muscle function. There was no difference in contraction time between the CON and CT groups after the casting period of 14 days. However, to our surprise, the application of the three treatments (CT-AC, CT-EA, and CT-ES) during the cast-immobilization period demonstrated a significant increase in contraction time (*p* < 0.05) compared to the CON and CT groups. Previous work employing the same treatment conditions during 14 days of cast-immobilization revealed no difference in contraction time when compared to both the control and cast groups [17]. Contradictory findings in contraction time have been reported, with both four weeks and six to eight weeks of casting reducing and increasing contraction time, respectively [30,31].

The present investigation showed a 44% reduction in peak twitch force after cast-immobilization lasting 14 days, aligning with Belova et al.’s [32] 38% decrease in twitch tension after seven days of mechanical unloading. Notably, the CT-AC, CT-EA, and CT-ES groups in the current study exhibited a significant treatment effect for peak twitch force and did not differ from the CON group. Of note, the current finding of no difference in peak twitch tension between the CT-AC and the CON group aligns with the result of Symons et al., who found the CON and CT-AC groups did not differ with respect to peak twitch tension after the cast-immobilization period of 14 days [17].

Following cast-immobilization, peak tetanic tension of the gastrocnemius muscle was diminished by 29%. This diminished peak tetanic tension demonstrated by the CT group was expected and aligned with previous research that demonstrated a 42% and 65% reduction in the plantaris and soleus muscles, respectively [17,32]. Once again, the CT-AC, CT-EA, and CT-ES groups in the current study exhibited a meaningful treatment effect for peak tetanic force as they did not differ from the CON group.

Importantly, our results, in conjunction with previous literature, found that acupuncture, electro-acupuncture, or electrical stimulation have a notable preventive influence on skeletal muscle contractile properties.

### 4.3. Biochemical Measures

In various models of skeletal muscle atrophy, two ubiquitin ligases, namely MAFbx and MuRF1, are upregulated. These ligases are accountable for elevated protein breakdown through the ubiquitin-proteasome [33,34,35]. The present study found that cast-immobilization for a period of 14 days increased MAFbx and MuRF1 by 169% and 87%, respectively. Our results align with previous work demonstrating elevated MAFbx and MuRF1 protein levels following five to seven days, 14 days, and five weeks of cast-immobilization [17,26,36]. Viewing these results collectively, casting and the subsequent reduction in skeletal muscle mechanical loading experienced by our animals led to an increase in skeletal muscle protein breakdown.

Exercise represents the most valid treatment option to counteract the deleterious effects of skeletal muscle atrophy [37,38]. However, it may not always be feasible for individuals who are bedridden or battling illness, necessitating the need for non-exercise-based interventions [24]. Importantly, the current findings show that acupuncture, electro-acupuncture, and electrical stimulation treatments preserved MAFbx and MuRF1 protein levels during the cast-immobilization period (14 days) when compared to baseline levels. Prior research supports our results; Onda et al. [13] demonstrated that 14 treatments of acupuncture and electro-acupuncture during hind limb suspension for 14 days significantly reduced soleus muscle MAFbx and MuRF1 mRNA expression compared to the group receiving only hind limb suspension. Symons et al. further established that acupuncture, electro-acupuncture, and electrical stimulation treatments preserved MAFbx and MuRF1 protein levels within the plantaris muscle during cast-immobilization for 14 days [17]. Taken as a whole, these findings suggest that acupuncture, electro-acupuncture, and electrical stimulation may serve as alternatives to pharmaceutical and exercise-focused interventions. They could also supplement existing interventions in the management of disused skeletal muscle loss. This is achieved by downregulating MAFbx and MuRF1 protein levels and mRNA expression, effectively attenuating skeletal muscle disuse and wasting. 

The impact of immobilization on plasma inflammatory cytokine levels was determined given the established association between muscle atrophy and elevated inflammation markers [39]. Contrary to expectations, our study found no significant differences in IL-6 levels between the studied groups. Limitations in assay sensitivity hindered our ability to reliably detect other inflammatory markers, including IL-1β, tumor necrosis factor-alpha (TNF-α), and monocyte chemoattractant protein-1 (MCP-1), in most plasma samples. The methodologies used in our study, specifically the casting methods, might not have been robust enough to elicit systemic and chronic inflammation. This limitation potentially obscured the detection of significant changes in inflammatory cytokines, contrasting with findings from studies using models like tibial nerve denervation, which effectively demonstrate atrophy [24].

Further research is needed, and one such area of investigation could include the influence of acupuncture and electro-acupuncture on the structure and function of the neuromuscular junction (NMJ) during skeletal muscle disuse. Key indicators of NMJ destabilization, such as the loss of low-density lipoprotein receptor-related protein 4 (LRP4), reduced glycosylation of α-dystroglycan, and the downregulation of acetylcholine receptors, are reflective of transmission malfunction at the NMJ, a contributing influence on skeletal muscle wasting [40,41,42,43]. While we recognize the significance of sarcolemma integrity markers such as dystrophin, dystroglycans, and sarcoglycans, incorporating these measurements would have required additional resources and methodologies beyond the scope of our current project. Similarly, we did not measure centralized nuclei and creatine kinase (CK) levels in this manuscript. These variables, while important for assessing muscle damage and regeneration, were outside the primary focus of our study. Our research was specifically designed to address other critical aspects of muscle physiology, focusing on contractile properties, muscle characteristics, and two key atrophic proteins. However, we acknowledge their importance and suggest that future projects should include these variables to enhance and expand upon our findings. Additionally, exploration of the effects of acupuncture and electro-acupuncture on the signaling pathways that promote skeletal muscle growth, such as the mammalian target of rapamycin complex 1 (mTORC1) pathway, an important activator of protein synthesis and preventer of autophagy, would expand on the current study’s findings.

## 5. Conclusions

In conclusion, our study found that acupuncture, electro-acupuncture, or electrical stimulation, administered during a period of cast-immobilization lasting 14 days, attenuated disuse skeletal muscle loss. We further demonstrated that all three treatments reduced the loss of peak twitch and peak tetanic tension after cast-immobilization. Our findings indicate that the application of acupuncture, electro-acupuncture, or electrical stimulation could serve as treatment approaches for mitigating skeletal muscle atrophy and functional impairment associated with disused skeletal muscle loss across various diseases and aging.

## Figures and Tables

**Table 1:** Body Weight.

Characteristics	Group				
	CON (*n* = 8)	CT (*n* = 8)	CT-AC (*n* = 8)	CT-EA (*n* = 8)	CT-ES (*n* = 8)
Pre Cast Body Weight (g)	203.1 ± 7.2	205.3 ± 16.8	207.6 ± 11.9	201.1 ± 13.0	209.5 ± 9.0
Post Cast Body Weight (g)	213.1 ± 6.2 *	203.4 ± 15.1	202.3 ± 9.2	197.5 ± 11.1	208.8 ± 9.2

Values are means and standard deviation. CON = control; CT = cast; CT-AC = cast + acupuncture; CT-EA = cast + electro-acupuncture; CT-ES = cast + electrical stimulation. * Difference in pre-cast body weight and post-cast body weight (*p* < 0.05).

**Table 2:** Gastrocnemius Muscle Characteristics.

Characteristics	Group				
	CON (*n* = 8)	CT (*n* = 8)	CT-AC (*n* = 8)	CT-EA (*n* = 8)	CT-ES (*n* = 8)
Muscle Weight (mg)	1149.6 ± 121.0	666.6 ± 60.2 *	788.0 ± 103.7 *†	759.2 ± 108.5 *	744.9 ± 64.2 *
Gastro/body, ×10^5^	539.1 ± 49.5	329.4 ± 38.1 *	389.9 ± 52.1 *	385.1 ± 56.4 *	357.1 ± 29.6 *
Lo (mm)	37 ± 2.0	35 ± 1.3	36 ± 2.1	34 ± 0.9	35 ± 1.1
Gastrocnemius CSA (mm^2^)	1.09	1.01	1.04	1.04	1.04
Gastrocnemius Fiber CSA (µm^2^)	17,748.0 ± 2698.5	7473.8 ± 1492.8 *	9215.2 ± 713.7 *†	8895.7 ± 807.4 *†	9188.3 ± 801.7 *†

Values are means and standard deviation. Lo = Muscle Fiber Length; CSA = cross-sectional area; CON = control; CT = cast; CT-AC = cast + acupuncture; CT-EA = cast + electro-acupuncture; CT-ES = cast + electrical stimulation. * Difference from CON group (*p* < 0.05). † Difference from CT group (*p* < 0.05).

**Table 3:** Contractile Properties of the Gastrocnemius Muscle.

Characteristics	Group				
	CON (*n* = 8)	CT (*n* = 8)	CT-AC (*n* = 8)	CT-EA (*n* = 8)	CT-ES (*n* = 8)
CT (ms)	26.14 ± 7.71	29.33 ± 6.37	34.13 ± 2.17 *	34.75 ± 3.88 *	37.00 ± 4.34 *
1/2 RT (ms)	24.43 ± 8.36	19.00 ± 3.02	24.63 ± 3.42	28.75 ± 7.48 †	26.63 ± 4.44
Pt (N)	4.89 ± 1.13	2.74 ± 0.33 *	3.74 ± 0.75 †	3.70 ± 0.54 †	3.98 ± 0.58 †
SPt (N/cm^2^)	4.2 ± 1.0	3.8 ± 0.7	4.5 ± 0.9	4.3 ± 0.6	4.8 ± 0.8 *
Po (N)	10.43 ± 3.20	7.46 ± 1.32 *	7.78 ± 1.64	8.49 ± 2.23	8.89 ± 1.97
SPo (N/cm^2^)	8.8 ± 2.3	10.1 ± 1.9	9.5 ± 2.4	10.3 ± 2.2	11.1 ± 2.6

Values are means and standard deviation. CT = contraction time; 1/2 RT = half relaxation time; Pt = peak twitch tension, Po = peak tetanic tension; SPt = specific peak twitch tension; SPo = specific peak tetanic tension; CON = control; CT = cast; CT-AC = cast + acupuncture; CT-EA = cast + electro-acupuncture; CT-ES = cast + electrical stimulation. * Difference from CON group (*p* < 0.05). † Difference from CT group (*p* < 0.05).

## Data Availability

The data will be made available by the corresponding author upon reasonable request.
